# Cold-Welding and Over-Welding in Polyethylene Pipe Butt Fusion Joints: Formation Mechanisms, Structure–Property Degradation, and Emerging Microwave-Based Detection Strategies

**DOI:** 10.3390/ma19194237

**Published:** 2026-10-06

**Authors:** Jiao Shi, Kexue Jing, Ningning Li, Gongtian Shen, Yong Pan

**Affiliations:** 1 School of Quality and Technical Supervision, Hebei University, Baoding 071000, China; 20249020005@stumail.hbu.edu.cn; 2 Hebei Special Equipment Supervision and Inspection Research Institute, Shijiazhuang 050061, China; 3 China Special Equipment Inspection and Research Institute, Beijing 100029, China

**Keywords:** polyethylene pipe, butt fusion joint, cold welding, over-welding, interfacial healing, thermo-oxidative degradation, microwave non-destructive testing, defect evaluation

## Abstract

**Highlights:**

**What are the main findings?**

- Two hidden PE weld defects have distinct mechanisms: cold welding = poor molecular interdiffusion; over-welding = thermal oxidation, chain scission, and microvoids.
- Microwave NDT offers a non-contact, dielectric-sensitive complement to conventional NDT; in reported studies, cold welding mainly distorts |S11|, while over-welding mainly attenuates |S21| and lowers the resonance Q factor.
- A structure–property–signal framework and three-level grading system are built; gaps and a roadmap for field microwave inspection are summarised.

**What are the implications of the main findings?**

- Visual and short-term pressure tests fail to detect hidden defects; balanced welding parameters are critical to avoid long-term brittle pipeline failure.
- Microwave non-contact inspection addresses a key limitation of conventional NDT for subtle interfacial and thermally degraded weld defects, provided that frequency, lift-off and calibration are controlled.
- The evaluation framework and roadmap guide standardised quantitative microwave testing and full-life safety management of PE pipe networks.

**Abstract:**

Polyethylene (PE) pipes are extensively used in gas- and water-distribution systems because of their corrosion resistance, toughness, and installation flexibility. However, the field reliability of PE pipeline systems often depends on the quality of butt fusion joints, whose integrity is highly sensitive to welding parameters and thermal history. Among process-induced defects, cold welding and over-welding are particularly critical because they are usually concealed, difficult to identify by visual inspection or short-term pressure testing, and strongly associated with long-term brittle failure. Existing reviews have discussed butt fusion joining or polymer non-destructive testing in a broad sense, yet a defect-oriented synthesis linking process deviation, interfacial evolution, material degradation, and microwave response remains limited. This review therefore focuses specifically on cold welding and over-welding as the two most safety-critical hidden defects in PE butt fusion joints. First, the paper summarises how insufficient heat input, poor wetting, and limited molecular-chain interdiffusion lead to weak interfacial healing and low effective entanglement density, whereas excessive heat input and unfavourable thermal history promote chain scission, thermo-oxidative degradation, abnormal recrystallisation, and microvoid formation. It then compares how these mechanisms influence tensile behaviour, slow crack growth resistance, and long-term service performance. On this basis, conventional radiography and ultrasonics, phased-array ultrasonics, nonlinear acoustics, and microwave non-destructive testing (MNDT) are critically compared. Particular emphasis is placed on the structure–property–signal pathway that links local perturbations in complex permittivity to changes in microwave scattering parameters, including |S11|, |S21|, phase drift, resonance shift, and quality-factor reduction. Finally, a hierarchical evaluation framework is proposed to connect microwave features with defect severity and engineering decisions, and the major barriers to wider engineering adoption—benchmark datasets, quantitative inversion, lift-off robustness, and standardisation—are discussed. By organising the field around a mechanism-based structure–property–signal framework, this review aims to provide a more rigorous basis for defect-oriented quality control and future intelligent inspection of PE butt fusion joints.

## 1. Introduction

### 1.1. Engineering Background and Joint Vulnerability

Polyethylene (PE) is a thermoplastic semi-crystalline polymer whose molecular backbone is primarily composed of long-chain saturated hydrocarbons. Owing to its excellent resistance to chemical corrosion, favourable low-temperature toughness, and good environmental stress-cracking resistance, high-density polyethylene (HDPE) and medium-density polyethylene (MDPE) pipes have been widely used in urban gas distribution, water-supply networks, and industrial fluid transport [1,2,3,4]. Compared with metallic pipelines, PE pipes offer clear advantages in corrosive service environments and under underground settlement or local impact because their flexibility can provide additional structural tolerance [2,3].

In engineering practice, medium- and large-diameter PE pipes are predominantly joined by butt fusion welding. Unlike metallic welding, this process does not rely on filler addition or metallurgical solidification; rather, it is governed by interfacial melting, wetting, molecular-chain interdiffusion, and recrystallisation across the fusion interface [3,4,5,6,7,8]. Field experience and accident analyses consistently show that failure seldom initiates in the parent pipe, but instead concentrates in the joint region, where the material experiences a complex thermo-mechanical cycle and develops microstructural and residual-stress characteristics distinct from those of the base material [5,6,7,8,9,10]. Accordingly, the welded joint is widely regarded as the weakest link controlling the long-term integrity of PE pipeline systems.

### 1.2. Concealed Defects and Long-Term Service Risk

The quality of a butt fusion joint depends strongly on the coordinated control of temperature, heating time, pressure, changeover, and cooling conditions [11,12,13,14,15,16]. When the process window is exceeded or poorly controlled, multiple defect modes may occur. Gross geometric defects such as obvious misalignment or severe bead irregularity can usually be recognised by visual examination. By contrast, cold welding and over-welding are far more problematic because they are often concealed, may pass short-term acceptance checks, and directly undermine the long-term crack resistance of the joint [11,12,13,14,17,18,19,20].

Cold welding is generally associated with insufficient heat input, incomplete wetting, limited diffusion depth, or inadequate effective contact time, which together prevent the formation of a sufficiently entangled interfacial network. Over-welding, in contrast, reflects excessive heat input or an unfavourable thermal history that promotes chain scission, oxidative embrittlement, abnormal recrystallisation, and internal voiding. Although the short-term manifestations of these two defects differ, both create weak zones that can accelerate slow crack growth (SCG), reduce creep rupture life, and ultimately trigger brittle failure under internal pressure, ground movement, or environmental loading [12,18,19,20,21].

### 1.3. Aim, Scope, and Positioning of This Review

The non-destructive evaluation of concealed PE weld defects remains a major challenge. Magnetic-particle and eddy-current methods are fundamentally unsuitable for non-conductive polymers; radiographic testing is insensitive to weak interfacial defects with limited density contrast; and conventional ultrasonic testing is constrained by high attenuation and scattering in PE [22,23,24,25,26,27,28]. Advanced acoustic approaches and microwave-based methods have therefore attracted growing interest, especially for detecting subtle interfacial degradation that cannot be judged reliably from external appearance alone [26,27,28,29,30,31,32,33,34,35,36,37].

Unlike broader reviews on PE pipe joining or general non-destructive testing, the present article is organised around a defect-oriented and mechanism-based question set: how welding-parameter deviation alters interfacial healing or thermal degradation; how these structural changes translate into mechanical deterioration; why conventional methods struggle to resolve such defects; and how microwave sensing can be used to build a hierarchical, risk-oriented evaluation framework. The goal is not merely to summarise previous studies, but to clarify the process–microstructure–property–signal linkage that is essential for publishable mechanistic interpretation and future field deployment.

Several earlier works define the starting point of this review. Alhatti et al. [3] reviewed fusion conditions and joint-integrity assessment for HDPE butt fusion with a process-technology emphasis; Wang et al. [38] provided a broad review of PE pipes covering failure mechanisms, performance models, inspection and repair; and Ghattas et al. [30] reviewed non-destructive microwave testing of material defects in general terms. These works are complementary but do not offer a defect-oriented synthesis that treats cold welding and over-welding as two distinct, competing degradation routes and carries each route from welding-parameter deviation through interfacial and chemical evolution to its mechanical and microwave signatures. Against that background, the specific contributions of this review are as follows: (1) a unified, mechanism-based comparison of under-healing and thermal damage within a single effective thermal-history framework; (2) a statement of the governing theory for interfacial healing kinetics and for the permittivity–S-parameter–Q relationship; (3) a critical, condition-documented comparison of representative NDT studies (Table 1), including frequency bands, sensors and reported limitations; and (4) a three-level signal–fracture-mode–action grading concept together with a field-deployment roadmap.

Three explicit propositions emerge from this synthesis and define the new understanding sought by this review: (i) Cold welding and over-welding are not mirror images but two competing failure routes—under-healing versus thermal damage—that can be ordered along a single effective thermal-history coordinate and that produce opposite dielectric perturbations (dominantly ε′/reflection versus ε″/loss). (ii) The reported microwave signatures become interpretable, rather than anecdotal, only when the chain welding history → interfacial/material state → ε′/ε″ perturbation → (|S11|, |S21|, phase, Q) is stated quantitatively; apparent disagreements between studies then largely resolve into differences in sensor type, frequency, lift-off and defect preparation. (iii) For engineering use, signal abnormalities must be fused with fracture-mode evidence and explicit rejection of confounding factors before being mapped to a service decision; this motivates the three-level grading concept and the standardisation roadmap.

Research in this area has continued to advance in 2026. Industrial computed tomography (CT) has been used to image PE butt joints produced at different welding temperatures, revealing characteristic white fusion-zone lines for cold welding (low-density unfused regions resulting from restricted chain interdiffusion) and black arc-shaped features for over-welding (thermal-oxidative degradation with volatile-induced porosity) [40]. Microwave line-scanning combined with hierarchical machine-learning recognition has been demonstrated on PE110 joints (7 mm wall): simulation over 12.4–18 GHz identified 15.984 GHz as the selected working frequency, and reflection-coefficient (|S11|) features classified cold weld, slag-inclusion and void defects [41]. A separate 2026 study systematically optimised the inspection frequency band through electromagnetic simulation of |S11| responses for PE pipe joints [42]. These recent works confirm both the rapid evolution of the field and the need, addressed here, for a mechanism-based and condition-aware interpretation of the signals.

### 1.4. Literature Search and Selection Strategy

This is a narrative (non-PRISMA) review, but the literature search was conducted systematically for transparency. We retrieved records from Web of Science, Scopus, and Google Scholar; located Chinese-language studies and theses through CNKI and Wanfang; and identified standards through ISO, ASTM, and Chinese national-standard portals, up to June 2026. Search strings combined (‘polyethylene’ OR ‘PE’ OR ‘HDPE’ OR ‘MDPE’) AND (‘butt fusion’ OR ‘thermal fusion’ OR ‘hot-melt’ OR ‘electrofusion’) AND (‘cold weld*’ OR ‘over-weld*’ OR ‘lack of fusion’ OR ‘defect*’ OR ‘microwave’ OR ‘ultrason*’ OR ‘NDT’ OR ‘NDE’). Peer-reviewed papers, conference papers, theses, and standards reporting process–structure, mechanical, or inspection evidence for PE pipe fusion joints were retained; records unrelated to PE pipe joints, duplicates, and items that could not be verified against publisher metadata were excluded. Titles and abstracts were screened first, followed by full-text assessment, and bibliographic details of retained records were cross-checked with publisher or Crossref records.

## 2. Butt Fusion Welding and the Microstructural Basis of Joint Integrity

### 2.1. Interfacial Healing Theory and Kinetics in Hot-Melt Welding

From the perspective of polymer physics, PE butt fusion is essentially an interfacial healing process. At room temperature, PE consists of crystalline lamellae embedded in an amorphous phase, and large-scale molecular-chain motion is strongly restricted. Once the pipe ends are heated above the melting range, the pre-existing crystalline structure collapses and the material transitions from an elastic solid into a viscous melt in which long-chain segments acquire substantial conformational freedom [1,3,13,43].

During alignment and joining, the two molten surfaces are brought into intimate contact. Molecular chains on both sides then reptate and interdiffuse across the original interface, progressively building a network of physical entanglements. During subsequent cooling, the interdiffused chains participate in crystallisation and generate a new crystalline–amorphous structure that can, under appropriate conditions, approach the continuity and load-transfer capability of the parent material [3,4,13]. The integrity of the final joint is therefore determined less by the disappearance of the visible interface than by the depth of interfacial healing and the preservation of molecular stability.

From a kinetic viewpoint, healing is commonly described in five stages: surface rearrangement, surface approach, wetting, diffusion and randomisation [44]. Within the reptation model, a chain escapes its original tube on the reptation (disengagement) time $\tau_{rep}$, which scales with molecular weight as shown in Equation (1) [45]. The recovering interfacial strength then follows Equation (2) for t < $\tau_{rep}$ and approaches the bulk value σ∞ once t ≳ $\tau_{rep}$ [46,47]. Cold welding corresponds to the regime in which the effective contact time at adequate melt temperature is too short (t < $\tau_{rep}$), or the molten layer is too thin, so that interpenetration depth and effective entanglement density remain below parent-material levels. These relations also explain why temperature, which controls chain mobility and hence τrep, cannot be compensated by pressure alone:

(1) $$\tau_{rep}\propto M^{3}$$

(2) $$\frac{\sigma (t)}{\sigma_{\infty }}\approx {\left(\frac{t}{\tau_{rep}}\right)}^{1/4},t<\tau_{rep}$$

### 2.2. Synergistic Effects of Thermo-Mechanical Parameters

The standard butt fusion procedure may be divided into end preparation, heating and melting, changeover and joining, and pressure-holding during cooling [14,15,16,48]. These operational stages translate into a coupled thermo-mechanical history controlled by heating temperature, heating time, pressure, changeover time, and cooling conditions. Temperature and heating time determine the thickness of the molten layer and the activation of chain mobility; pressure affects wetting, squeeze flow, and retention of high-quality molten material in the weld core; and cooling governs recrystallisation kinetics and the timing at which interfacial diffusion is arrested.

The relationship among these parameters is strongly nonlinear. Insufficient heating produces only a thin molten layer and shallow diffusion depth, whereas excessively high temperature or prolonged heating increases the risk of oxidation, chain scission, and volatile evolution. Pressure is similarly double-edged: inadequate pressure limits intimate contact, while excessive pressure can expel too much molten material into the bead and leave the weld core locally depleted. Cooling that is too short or poorly controlled may freeze the interfacial state before a sufficiently robust entangled network develops. In practice, defect formation should therefore be interpreted as the outcome of an imbalanced process window rather than a single-parameter deviation.

### 2.3. Hierarchical Structure of the Weld Region

After butt fusion, the joint typically exhibits a heterogeneous through-thickness and axial structure comprising the parent-material zone (PM), the heat-affected zone (HAZ), and the melt-fusion zone (MFZ). The PM largely retains the original structure, the HAZ experiences thermal exposure without complete melting, and the MFZ undergoes full melting and resolidification [4,11,49]. In an ideal MFZ, a continuous crystalline–amorphous network is rebuilt across the former interface, allowing the joint to combine stiffness, strength, and plastic energy dissipation.

**Figure 1:**
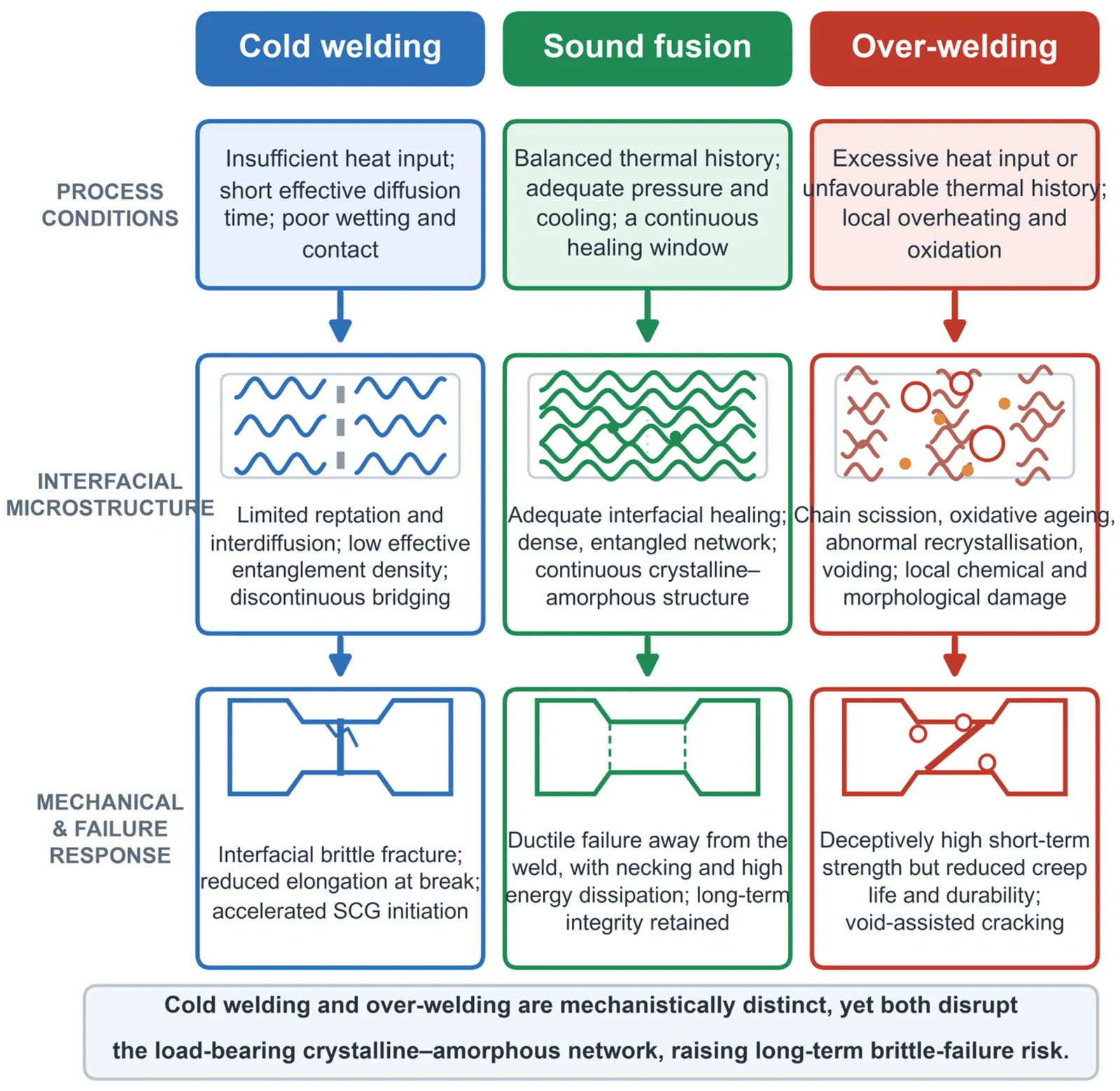
Schematic comparison of process–microstructure–failure pathways for cold welding, sound fusion and over-welding in PE butt fusion joints. Wavy lines represent polymer molecular chains; circles and dots denote structural defects and oxidative by-products. The three columns correspond to cold welding (blue box), sound fusion (green box), and over-welding (red box), respectively. Original schematic prepared by the authors; the molecular-scale healing and thermal-damage concepts are synthesised from classical polymer-interface healing theory [44,45,46,47] and standard butt-fusion references [1,13,49].

A sound joint is therefore characterised by two essential structural features: continuity of the crystalline skeleton across the former interface and a sufficiently dense entangled network within the amorphous phase. Process deviations that disturb either one of these features can generate latent damage even when the external bead appears acceptable. Figure 1 summarises this process–microstructure–failure logic and highlights why cold welding and over-welding should be treated as distinct mechanistic routes to long-term performance degradation.

## 3. Formation Mechanisms and Mechanical Implications of Representative Defects

### 3.1. Cold Welding: Insufficient Interfacial Healing

Cold welding is one of the most concealed weak-bond defects in PE butt fusion joints. Its core feature is not simply low heat input in a macroscopic sense, but incomplete interfacial healing at the molecular scale. When thermal and contact conditions are insufficient, the molten layers fail to wet and interpenetrate adequately; the diffusion depth remains shallow, and the density of effective entanglements across the interface stays well below that required for parent-material-like integrity [2,11,12,13,14,50]. Fractographic observations reported in the literature often show unfused boundaries, island-like discontinuities, or markedly shortened craze fibrils, all of which are consistent with restricted plastic energy dissipation and premature interfacial separation.

Insufficient cooling is not a single mechanism and should be distinguished into (i) premature pressure release or unloading before the joint cools below the handling temperature, when shrinkage stresses can re-open the partially formed interface; (ii) rapid or forced cooling (quenching), which produces non-uniform crystallisation, elevated residual stress and a frozen-in under-entangled skin; and (iii) early handling or movement before sufficient rigidity, causing mechanical disturbance or micro-misalignment of the interdiffusing interface.

### 3.2. Over-Welding: Thermal Degradation and Microvoid Formation

Over-welding is mechanistically different. In this case, the issue is not insufficient healing, but excessive thermal exposure or an unfavourable thermal history that destabilises the polymer itself. Prolonged or excessive heating may accelerate oxidative reactions, alter molecular-weight distribution, and increase the probability of chain scission. During subsequent cooling, this degraded melt may recrystallise abnormally, while low-molecular-weight products or entrapped gases can promote microvoid or bubble formation in the MFZ or adjacent HAZ [12,17,18,19,30,40].

An important reason why over-welding is difficult to detect in the field is that it can be deceptively benign in short-term appearance or even in short-term strength. A wide and apparently well-formed bead does not necessarily imply a robust molecular structure. In many cases, the dominant penalty of over-welding emerges not as immediate gross fracture, but as reduced oxidation resistance, higher local defect density, faster SCG initiation, and a shorter creep life under prolonged service loading [18,19,20,21].

### 3.3. Welding-Induced Versus Service-Induced Degradation

Thermal-oxidative degradation in PE joints arises from two regimes that should not be conflated. Welding-induced degradation occurs during a short, high-temperature excursion, in which the melt is held above the melting range for seconds to minutes: locally within the MFZ and adjacent HAZ, limited oxygen access, chain scission, abnormal recrystallisation and volatile- or shrinkage-driven microvoids are generated, and the damage is already present in the as-welded state—for example as dark CT features or a reduced oxidation induction time immediately after welding [40].

Service-induced degradation instead develops over years at much lower temperatures, under the combined action of oxygen, ultraviolet exposure, disinfectants such as chlorine dioxide, internal pressure and soil loading; it progresses from the exposed surfaces inward, affects the pipe wall more broadly, and is commonly studied through natural or accelerated thermal, hydrothermal or photo-ageing [18,51,52,53]. Accelerated-ageing experiments therefore simulate the latter regime rather than measure welding damage. The two regimes share chemistry but differ in time scale, spatial distribution and driving variables; over-welding is additionally consequential because it consumes part of the antioxidant reserve and can thereby accelerate—and be mistaken for—later in-service ageing. Attribution in the field should consequently combine welding records, joint age and the spatial pattern of damage.

### 3.4. From Local Damage to Macroscopic Failure

Despite their different origins, cold welding and over-welding converge in one crucial respect: both interrupt the load-bearing crystalline–amorphous network that gives a high-quality PE joint its long-term integrity. Cold welding does so by leaving the interface under-healed and mechanically discontinuous, whereas over-welding does so by degrading the material and creating chemically and morphologically weakened zones. As a result, both defects can shift tensile failure from ductile deformation in the parent material to premature fracture in or near the weld, with associated losses in elongation at break and fracture toughness [11,12,20,21,49].

**Figure 2:**
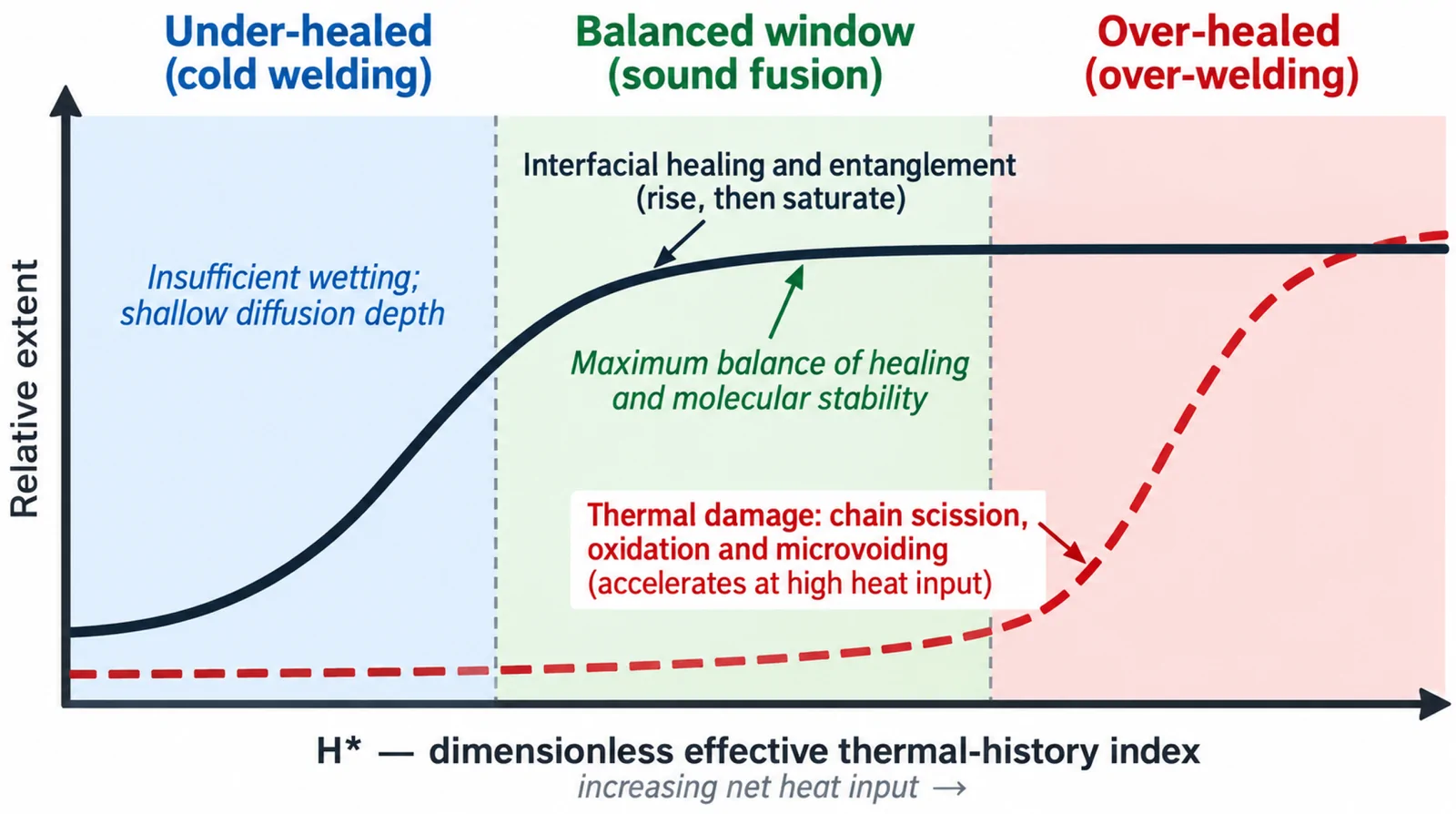
Schematic, non-quantitative illustration of the competition between interfacial healing and thermal damage across the butt fusion process window. The curves express qualitative trends only; they are not fitted to experimental data and deliberately carry no numerical scale. The horizontal coordinate is a dimensionless effective thermal-history index (defined in Section 3.4) that increases with net heat input; it serves only to order the under-heated (cold welding), balanced (sound fusion) and over-heated (over-welding) regimes.

A useful way to conceptualise these mechanisms is to consider defect evolution as a competition between interfacial healing and thermal damage. At low effective heat input, the interfacial network never matures fully; at excessively high heat input, material degradation increases more rapidly than any further gain in healing. Figure 2 presents this competition schematically and explains why the practical process window must balance adequate molecular interdiffusion with the preservation of molecular stability.

To make the conceptual nature of Figure 2 explicit, the horizontal coordinate is defined as a dimensionless effective thermal-history index, Equation (3), where T is the interfacial temperature, Tm the melting range, tc the effective contact time in the melt, τrep the reptation time, and P the joining pressure; f, g, and h denote monotone shape functions and wT, wt, wP arbitrary positive weights. No numerical values are assigned to the weights or to H*, and the healing and damage curves are qualitative monotone trends—healing rises with H* and saturates, whereas thermal damage accelerates at high H*. Their crossover delineates only, in order, an under-healed regime (cold welding), a balanced window (sound fusion, conceptually consistent with the parameter windows of ASTM F2620 and ISO 21307 [14,15,54]) and an over-heated regime (over-welding). Figure 2 must not be read as a fitted or predictive model; quantitative versions would require grade-specific thermal and kinetic data and are identified as an open need in Section 6.

(3) $$H^{*}=w_{T}f(T-T_{m})+w_{t}g\left(\frac{t_{c}}{\tau_{rep}}\right)+w_{P}h(P)$$

## 4. State of the Art in Defect Detection for PE Butt Fusion Joints

### 4.1. Limitations of Conventional Non-Destructive Testing

Traditional non-destructive testing methods do not map neatly onto PE weld inspection. Magnetic-particle and eddy-current testing are inapplicable because PE is neither ferromagnetic nor electrically conductive. Radiographic testing can identify sufficiently large volumetric discontinuities but offers poor sensitivity to cold-welded interfaces because such defects may involve only limited density variation. Conventional pulse-echo ultrasonics has broader applicability, yet PE exhibits relatively high acoustic attenuation and strong scattering, both of which reduce spatial resolution and make weak interfacial defects difficult to discriminate with confidence [22,23,24,25,26,27,28].

### 4.2. Advanced Acoustic Methods

To address these challenges, phased-array ultrasonic testing (PAUT) and nonlinear ultrasonic approaches have been explored for PE pipe joints [26,27,28]. PAUT improves beam control through electronic focusing and steering, which can enhance localisation of gross lack-of-fusion regions or void-rich zones when array frequency and coupling conditions are appropriately selected. Nonlinear acoustics is even more attractive from a damage-sensitivity perspective because higher-order harmonic generation can reveal microstructural nonlinearity associated with early-stage defects. However, both approaches still depend on robust acoustic coupling, are sensitive to surface condition and geometry, and can face practical limitations in scanning efficiency, interpretability, or field robustness.

### 4.3. Advances of Microwave NDT

Microwave non-destructive testing (MNDT) offers a fundamentally different sensing route because it interrogates the dielectric response of the material rather than its density contrast or acoustic impedance. For non-polar polymers such as PE, small perturbations in crystallinity, interfacial discontinuity, oxidation state, moisture uptake, or void content may alter the local complex permittivity and consequently influence reflected and transmitted electromagnetic signals [29,30,31,32,33,34,35,36,37,42,55,56,57]. This feature makes MNDT especially relevant for hidden defects that are structurally subtle but electromagnetically meaningful.

The principal attraction of MNDT for PE butt fusion joints is therefore not simply that it is non-contact, but that its observables can be related to the underlying defect physics. Cold welding tends to generate discontinuities in the real part of permittivity owing to interfacial gaps or insufficiently healed regions, while over-welding can increase dielectric loss through chemical ageing and defect-assisted energy dissipation. Table 1 summarises the relative capabilities and limitations of representative NDT methods for this application.

Methodologically, microwave inspection of PE fusion joints has evolved from early single-frequency reflectometry and open-ended waveguide probes applied to cold-fusion detection [34,56], through swept-frequency |S11| measurements [35] and microwave imaging [55], toward resonant-cavity/focusing-antenna probes operating near 23.14 GHz that track resonance frequency and |S21| [2,36], millimetre-wave FMCW imaging at 120 GHz [57], and machine-learning-assisted line-scan recognition [41]. Table 2 summarises representative studies with their test conditions and stated limitations.

Three cautions follow from this comparison. First, the reported working frequencies span roughly 9.9–120 GHz and are sensor- and defect-scale-specific rather than competing ‘best’ frequencies; higher frequency improves spatial resolution but reduces penetration. Second, reflection/|S11| studies emphasise interfacial gaps, whereas resonant |S21|/Q studies emphasise dielectric loss and inclusions, which explains the different signature assignments rather than contradicting them. Third, developer-led studies report very high sensitivity, whereas an independent evaluation of microwave imaging on butt-fusion joints found a good probability of detection but a false-call rate of about 57% on apparently sound joints [39]; most studies also use drilled, cut or artificially seeded defects rather than genuine process-induced cold- or over-welded joints. Sensitivity rankings are therefore conditional, and calibrated reference standards, probability-of-detection data and acceptance thresholds—not raw sensitivity—are the current bottleneck (Section 6). Accordingly, microwave methods should be viewed as complementary to acoustic and radiographic techniques: they are particularly sensitive to subtle interfacial and dielectric-loss defects under controlled frequency and lift-off, whereas acoustic methods remain preferable for certain volumetric and geometry-controlled tasks.

### 4.4. Microwave–Dielectric Interaction: Theoretical Basis

The dielectric state of a polymer is described by the complex permittivity and the loss tangent in Equation (4); ε′ denotes polarisation and energy storage, and ε″ denotes dielectric loss. For a plane wave at normal incidence on the interface of two low-loss dielectric media, whose intrinsic impedance and normal-incidence reflection coefficient are given by Equations (5) and (6); under matched-port conditions the measured reflection scattering parameter satisfies |S11| ≈ |Γ|, whereas transmission over a path of length d decays according to Equation (7), with the attenuation coefficient α increasing with ε″. For PE (εr ≈ 2.3–2.6 with very small ε″), an interfacial air gap (εair ≈ 1) produces a strong local impedance contrast and therefore perturbs mainly |S11| and the reflected phase.

(4) $$\varepsilon^{*}(\omega )=\varepsilon^{\prime }(\omega )-j\varepsilon^{\prime \prime }(\omega ),tan\delta =\frac{\varepsilon^{\prime \prime }}{\varepsilon^{\prime }}$$

(5) $$\eta_{k}=\sqrt{\frac{\mu_{k}}{\varepsilon_{k}}}$$

(6) $$\Gamma =\frac{\eta_{2}-\eta_{1}}{\eta_{2}+\eta_{1}}$$

(7) $$\left|S_{21}|\propto exp(-\alpha d\right)$$

When a resonant cavity or resonator is used, a small non-magnetic sample placed in the electric-field region perturbs the resonance according to Equation (8), with the quality factor defined by Equation (9); in the standard cavity-perturbation form, the real and imaginary relative permittivities follow Equations (10) and (11), where subscripts c and s denote the empty and sample-loaded cavity, and Vc, Vs denote the corresponding effective volumes [31]. Oxidation, moisture uptake or microvoid-assisted loss therefore appear predominantly as |S21| attenuation, resonance shift and Q reduction. These relations provide the physical basis for assigning cold welding mainly to reflection anomalies and over-welding mainly to transmission/loss anomalies in Section 5.

(8) $$\frac{f_{c}-f_{s}}{f_{s}}=A(\varepsilon^{\prime }-1),\Delta \left(\frac{1}{Q}\right)=2B\varepsilon^{\prime \prime }$$

(9) $$Q=\frac{f_{0}}{\Delta f_{3dB}}$$

(10) $$\varepsilon_{r}^{\prime }=1+\frac{V_{c}(f_{c}-f_{s})}{2V_{s}f_{s}}$$

(11) $$\varepsilon_{r}^{\prime \prime }=\frac{V_{c}}{4V_{s}}\left(\frac{1}{Q_{s}}-\frac{1}{Q_{c}}\right)$$

## 5. Microwave-Response Mechanisms and Hierarchical Defect Evaluation

### 5.1. Dielectric Perturbation Induced by Cold Welding and Over-Welding

For MNDT to move beyond qualitative anomaly detection, the electromagnetic response must be interpreted through a mechanism-aware lens. In cold-welded joints, the principal perturbation arises from interfacial structural discontinuity: incomplete wetting and insufficient chain interpenetration create local heterogeneity that alters the real component of complex permittivity and intensifies reflection-related anomalies. In over-welded joints, by contrast, thermal degradation, oxidation, and voiding may produce stronger changes in dielectric loss, resonance damping, and transmission behaviour [30,34,35,36,37,42,55,56,57,58,59,60,61,62].

These two response patterns are conceptually distinct even when they coexist in a complex real joint. Cold welding is more likely to manifest as strong |S11| contrast, phase clustering, or spatially localised reflection anomalies, whereas over-welding tends to be associated with |S21| attenuation, broader resonance deterioration, or quality-factor reduction. Table 3 compares the two defect types from the perspectives of cause, physical essence, typical micro-features, mechanical degradation, and microwave signature.

### 5.2. Multi-Dimensional Microwave Features and Rejection of Confounding Factors

A single microwave indicator is rarely sufficient for reliable engineering classification. Reflection amplitude alone may be influenced by lift-off, local geometry, or bead morphology; transmission changes may be diluted when the defect zone is shallow; and phase behaviour can be sensitive to both material state and measurement configuration. Robust interpretation therefore requires multi-dimensional features, including amplitude, phase, resonance position, bandwidth, and spatial continuity across scanning images. The most promising strategy is not to search for one universal feature, but to combine complementary observables that respond differently to interfacial discontinuity and thermal degradation.

Detection performance is governed by four groups of factors. (1) Defect characteristics: the dielectric contrast (air ε ≈ 1 versus PE ε ≈ 2.3–2.6; oxidation- and moisture-related increases in ε″), the defect dimension relative to the wavelength and sensing depth, and its orientation relative to polarisation and beam axis; thin, well-bonded weak interfaces remain the hardest target. (2) Frequency: higher frequency improves spatial resolution but reduces penetration depth and increases sensitivity to surface roughness, so the optimum frequency is pipe- and defect-specific (reported optima span roughly 16–24 GHz for wall-thickness-scale joints, with 120 GHz systems trading penetration for resolution). (3) Inspection geometry: lift-off variation, pipe curvature, bead topography and temperature all modulate the baseline signal. (4) Sensor configuration: open-ended waveguide, resonant cavity, imaging array and FMCW systems measure different observables with different noise floors. Accordingly, ‘very high’ sensitivity holds only within a matched defect–frequency–configuration envelope; outside it, acoustic or radiographic methods can be superior.

Because no single indicator is specific to a defect, the measured responses must be separated from four groups of confounding variations. (i) Lift-off: resonant probes operate at a fixed focal stand-off (e.g., 50 mm in recent 23.14 GHz systems [2,36]); constant-stand-off fixturing, a recorded lift-off–amplitude calibration curve and differential scanning against a defect-free reference zone suppress distance effects. (ii) Bead geometry: the bead generates a reproducible, symmetric signature; baseline subtraction, symmetric scanning, 360° circumferential normalisation and image registration distinguish it from genuine defects, which appear as localised, spatially continuous anomalies. (iii) Material composition: lot-to-lot differences in carbon-black dispersion, density, crystallinity and moisture shift the baseline permittivity [63]; same-joint parent-material reference zones and lot-level ε′/ε″ calibration are therefore required. (iv) Sensor configuration: multi-frequency and, where available, dual-polarisation measurements combining |S11|, |S21|, phase, resonance frequency and Q improve specificity, exploiting the fact that real defects extend coherently along the weld in scan images whereas lift-off and handling noise are spatially random. Fusion with acoustic, visual or geometric channels, and classifiers trained on data that explicitly include these confounders, provide a further safeguard [41].

**Figure 3:**
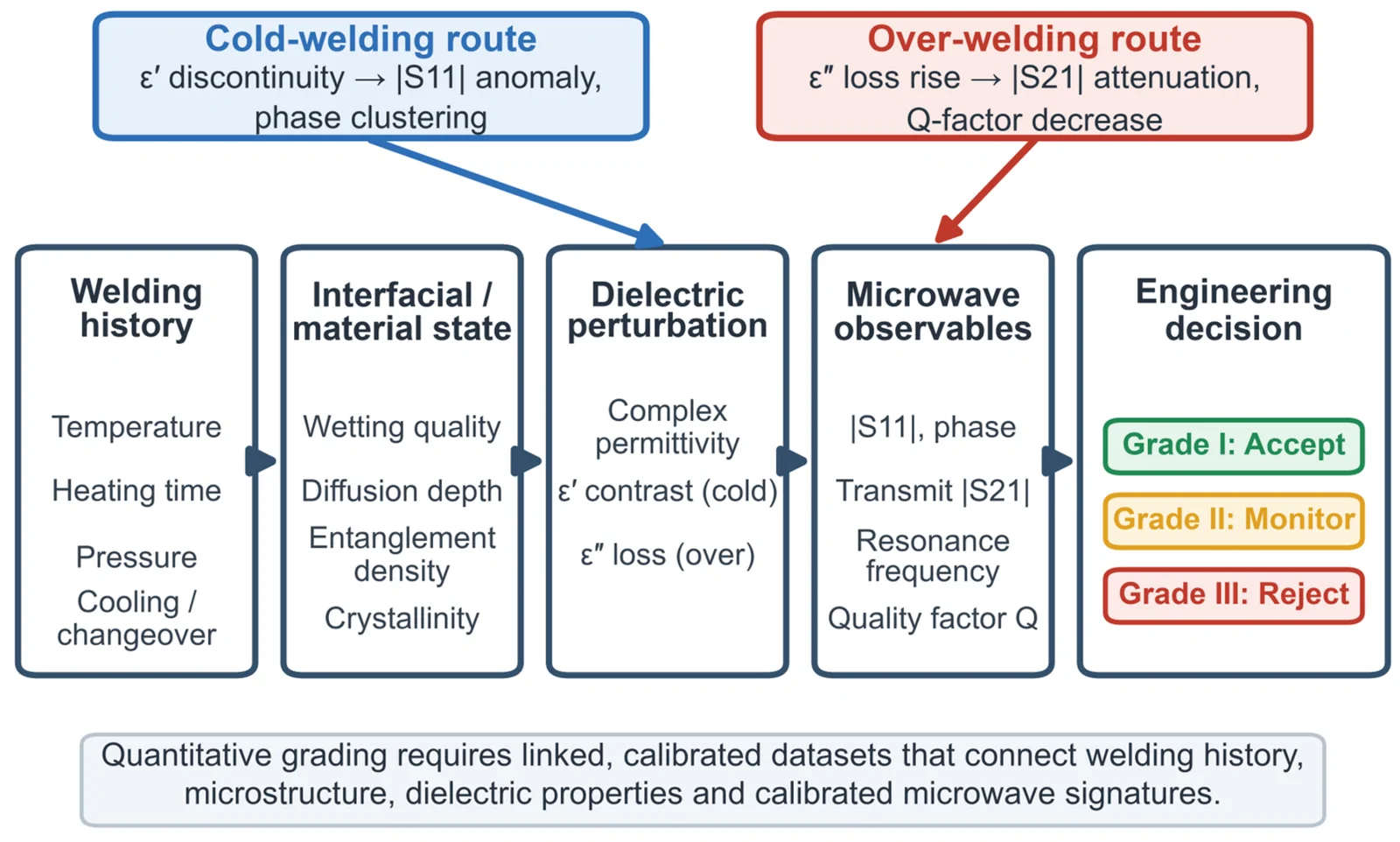
Structure–property–signal framework linking welding history, material perturbation, microwave observables, and engineering decisions.

Figure 3 illustrates the broader structure–property–signal framework that should guide this process. In a publishable and defensible workflow, welding history determines the interfacial state; the interfacial state governs material perturbation and complex permittivity; dielectric perturbation shapes the microwave observables; and the combined signal features are finally mapped to engineering decisions. This logic is essential if microwave inspection is to progress from attractive laboratory demonstrations to quantitative defect assessment.

### 5.3. From Signal Abnormality to Engineering Decision

From an engineering point of view, the real value of MNDT lies in defect grading rather than in simple defect presence/absence statements. A practical hierarchy should distinguish at least three levels: joints with no meaningful anomaly and acceptable long-term service potential; joints that show moderate abnormality and warrant monitoring or further confirmation; and joints whose signal characteristics indicate severe cold welding, deep thermal degradation, or a high probability of premature failure. Such a framework becomes much more persuasive when linked explicitly to mechanical evidence, fracture behaviour, and acceptance logic rather than to signal thresholds alone.

Table 4 presents a concise hierarchical evaluation concept that links microwave features with tensile fracture mode and engineering action. Although the exact thresholds must be established experimentally for specific pipe grades, geometries, and sensor configurations, the table provides a useful conceptual template for translating signal interpretation into service-oriented decisions.

## 6. Research Gaps and Future Directions

### 6.1. Quantitative Defect Inversion and Benchmark Datasets

A persistent weakness in the current literature is that many microwave studies remain proof-of-concept investigations with limited calibration depth. The field still lacks sufficiently broad benchmark datasets that connect controlled welding-parameter deviations with quantitative measurements of microstructure, dielectric properties, tensile behaviour, creep resistance, and microwave signatures. Without such linked datasets, it is difficult to determine whether a given anomaly reflects a true defect state, benign process variability, or measurement artefact. Building mechanism-grounded benchmark libraries should therefore be treated as a priority rather than as an auxiliary task.

### 6.2. Robust Field Deployment and Multi-Sensor Fusion

A second challenge is field robustness. Laboratory measurements are often obtained under controlled lift-off, surface cleanliness, and specimen curvature, whereas real PE pipeline joints may present irregular bead geometry, environmental variability, positional uncertainty, and access constraints. Future work should therefore focus on lift-off compensation, curved-surface adaptability, probe and fixture optimisation, and physics-informed multi-frequency inversion. The combination of microwave sensing with acoustic, visual, or geometric information is also likely to be valuable, because different modalities capture complementary aspects of defect formation and can improve classification robustness under realistic inspection conditions.

### 6.3. Standardisation, Validation, and Engineering Acceptance

The third major barrier concerns standardisation and validation. To gain wider acceptance in the pipeline sector, MNDT must be supported by reproducibility studies, probability-of-detection analysis, uncertainty quantification, and clearly defined acceptance criteria linked to service risk. Standard test blocks, reporting protocols, and cross-laboratory comparison exercises will be particularly important. More broadly, the field should avoid framing progress solely in terms of higher signal sensitivity; the more meaningful objective is reproducible and explainable defect quantification that remains valid across pipe grades, joint geometries, and inspection environments.

**Figure 4:**
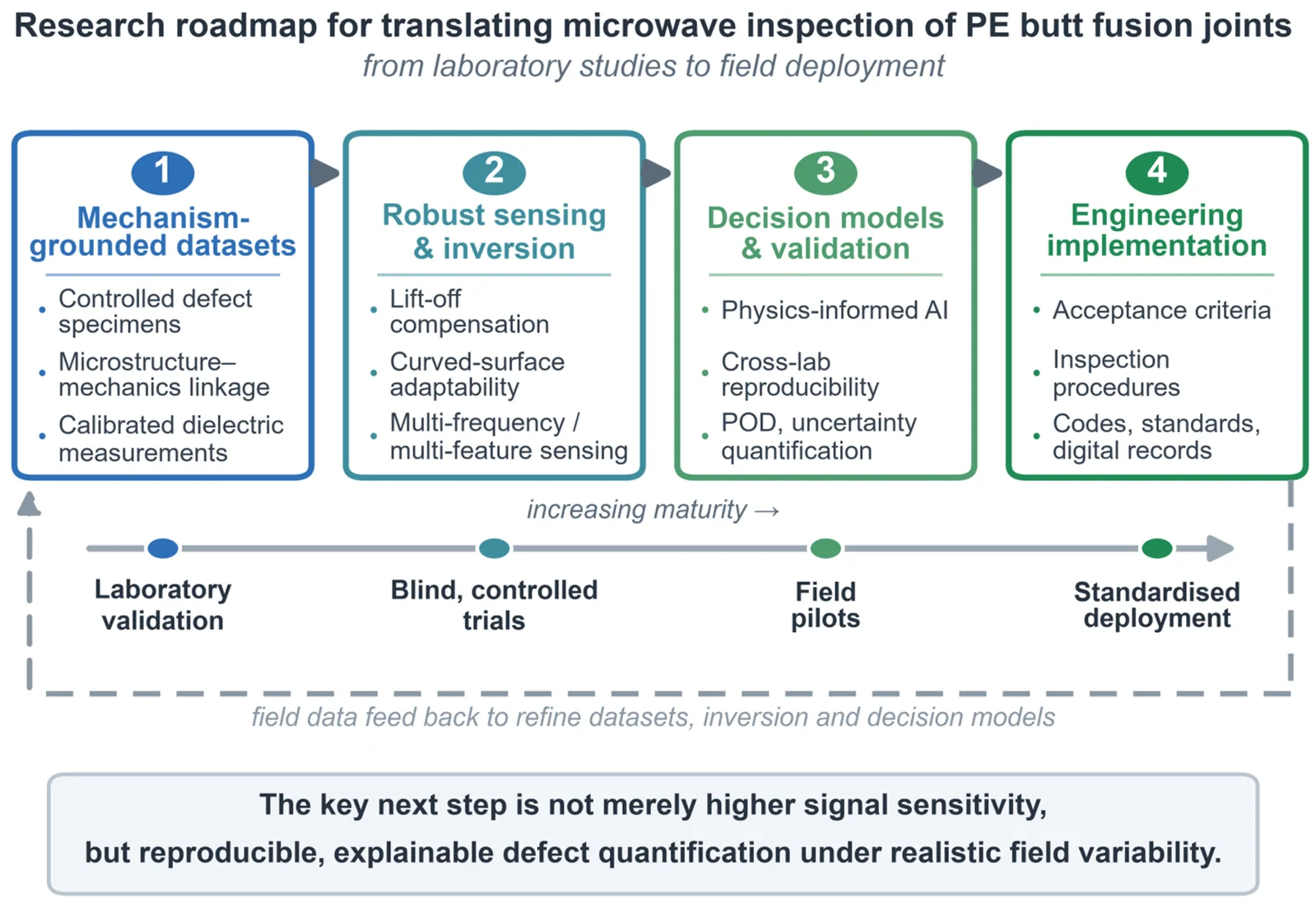
Research roadmap for translating microwave inspection of PE butt fusion joints from laboratory studies to field deployment.

Figure 4 summarises a pragmatic roadmap for advancing microwave inspection from laboratory studies to field deployment. The central message is that the next high-impact contribution will likely come from mechanism-grounded validation and decision reliability, not from isolated signal demonstrations alone.

## 7. Conclusions

Cold welding and over-welding represent two of the most safety-critical hidden defects in PE butt fusion joints, but they should not be treated as mirror images of the same problem. Cold welding is fundamentally a failure of interfacial healing, characterised by insufficient wetting, shallow diffusion depth, and low effective entanglement density. Over-welding, in contrast, is a degradation-driven defect associated with excessive thermal exposure, oxidative damage, molecular-weight redistribution, and microvoid formation. Their short-term manifestations may differ, but both disrupt the crystalline–amorphous load-transfer network that governs long-term performance.

The most valuable insight emerging from the recent literature is that reliable evaluation of these defects requires a linked process–microstructure–property–signal perspective. Microwave methods are promising precisely because they can respond to subtle dielectric perturbations associated with hidden weak-bond defects; however, their real engineering value will depend on whether they can be calibrated against defect physics, mechanical consequence, and decision thresholds. Future work should therefore prioritise benchmark datasets, robust inversion under field variability, and standardised validation frameworks. A mechanism-based, multi-scale evaluation strategy will be essential for improving weld quality control and long-term safety management in PE pipeline systems.

## Figures and Tables

**Table 1:** Comparative capabilities and limitations of representative non-destructive testing methods for concealed PE butt fusion-joint defects.

NDT Method	Sensitivity to Cold Welding	Sensitivity to Over-Welding	Penetration and Resolution	Contact Requirement	Core Limitations
Radiographic testing (RT)	Very Low	High (for macro-voids)	High penetration, low contrast	Non-contact	Radiation hazard, insensitive to interface gaps
Conventional ultrasonic (UT)	Low	Medium	High attenuation, low resolution	Requires couplant	High signal attenuation, prone to false negatives
Phased array ultrasonic (PAUT)	Medium	High	Balanced penetration and resolution	Requires couplant	Strict surface coupling requirements
Nonlinear acoustics	High	High	High sensitivity to micro-damage	Requires couplant	Complex signal processing, low scanning speed
Microwave NDT (MNDT)	High under optimised frequency/lift-off	High under optimised frequency/lift-off	Depth vs. resolution trade-off	Non-contact	Susceptible to lift-off effect

Note: qualitative ratings synthesised from the cited studies; sensitivity is defect-, frequency-, geometry- and configuration-dependent and is not a universal ranking. Independent trials reported an elevated false-call rate on apparently sound butt joints [39].

**Table 2:** Critical comparison of representative experimental studies on microwave and advanced inspection of PE fusion joints.

Reference (Year)	Material/Pipe	Defect Type (How Produced)	Method/Sensor	Frequency Band	Main Findings	Reported Limitations
Stakenborghs & Little (2009) [34]	HDPE butt-fusion joints	Cold fusion/lack of fusion, inclusions (process joints)	Single-frequency microwave imaging (open-ended probe)	~24 GHz class	Cold fusion detected and correlated with destructive tests	Independent field-style trials reported elevated false-call rates; geometry/coupling sensitive
Pan, Zhu, and Tan (2013) [35]	HDPE thermal-fusion welds	Cold welds (controlled welding conditions)	Swept-frequency reflection, |S11|	9.3–9.9 GHz	|S11| distinguishes cold welds from sound welds at the most sensitive frequency	Laboratory coupons; limited sample set; no over-welding
Wang et al. (2025) [2]	PE100, DN200 butt joints	Cold welds (fused at 170–230 °C)	Resonant cavity + focusing antenna, |S21|	23.14 GHz (penetration claimed >9 cm)	Welds below ~210 °C show significant resonance changes; crystallinity 88.4%→78.7%; tensile/toughness loss confirmed	Over-welding not covered; thresholds grade- and setup-specific
Pan, Zhu, and Tan (2025) [37]	PE100, DN200 butt joints	Holes (1–5 mm), cracks (1.5–4 mm deep), talc/sand inclusions (artificial)	Microwave resonant probe, |S21| and resonance shift	23.14 GHz; 50 mm stand-off	Dip magnitude scales with defect size; inclusions differentiated	Mainly artificial surface defects; integration level for field use pending
Li et al. (2026) [41]	PE110, 7 mm wall	Cold weld, slag inclusion, void (simulated/joint samples)	Microwave line-scan, |S11| + hierarchical ML recognition	Simulated 12.4–18 GHz; 15.984 GHz selected	Multi-defect classification from 18 selected time/frequency features	Simulation-heavy; field validation and generalisation ongoing
Sun et al. (2025) [57]	PE hot-melt joints (simulated)	Simulated internal defects	120 GHz FMCW millimetre-wave imaging	120 GHz	Internal imaging of hot-melt joints demonstrated	Resolution–penetration trade-off; limited field data
Hu et al. (2026) [40]	PE butt joints at varied welding temperatures	Cold vs. over-welding (process joints)	Industrial X-ray CT	X-ray CT (not microwave)	White fusion lines (cold, low-density unfused) vs. black arcs (over, oxidation/voids)	Bulky equipment, radiation safety, not readily field-deployable
Zhang et al. (2025) [8]	HDPE butt-fusion joints	Joint defects (process/seeded)	Phase-coherence TFM ultrasonics + improved YOLOX	Ultrasonic (acoustic benchmark)	Automated ultrasonic defect detection and localisation	Requires coupling; included as an acoustic comparator

**Table 3:** Mechanistic comparison between cold welding and over-welding, together with representative microwave-response signatures.

Defect Type	Core Causes	Physical Essence	Typical Micro-features	Mechanical Degradation	Dielectric Response Mechanism	Typical Microwave Signals
Cold welding	Low heating temperature; insufficient heating time; excessively high (or low) welding pressure; inadequate cooling-stage control, comprising (i) premature pressure release before cooling below the handling temperature, (ii) rapid/forced cooling (quenching), and (iii) early handling or movement (see Section 3.1).	Insufficient interfacial interdiffusion, low effective molecular entanglement density.	Presence of unfused physical interfaces, significantly shortened or missing craze fibrils on the fracture surface.	Prone to interfacial brittle fracture, sharp drop in yield strength and elongation at break.	Structural dielectric discontinuity (local abrupt change in the real part of permittivity, ε’).	Significant distortion in reflection coefficient amplitude |S11| and nonlinear phase drift or clustering.
Over-welding	Excessively high heating temperature, overly prolonged heating time, out-of-control cooling conditions (extremely slow heat dissipation).	Random scission of the carbon-carbon main chain, thermal-oxidative degradation of the material.	Destruction of the molecular weight distribution, leading to abnormal crystallisation and internal microvoid precipitation induced by thermal degradation.	Highly deceptive initial tensile strength but drastically reduced long-term creep life (SCG).	Chemical dielectric-loss amplification (increase in the loss factor, ε″).	Significant attenuation in transmission coefficient amplitude |S21| and a decrease in resonant quality factor (Q).

**Table 4:** Conceptual hierarchical evaluation framework linking microwave features, fracture behaviour, and engineering decisions.

Evaluation Grade	Defect State	Microwave Features (|S11|, |S21|, Phase)	Fracture Mode (Tensile Test)	Engineering Decision
Grade I (Safe)	Qualified joint/Slight fluctuation	Slight fluctuations, no continuous abnormal zones	Ductile failure in base material, long craze fibrils	Acceptable for long-term service
Grade II (Requires Monitoring)	Moderate cold welding/Early over-welding	Discontinuous abnormal zones, significant phase drift	Mixed type stripping along weld seam	Requires close monitoring, reduced service life expectancy
Grade III (High Risk)	Severe cold welding/Deep thermal-oxidative degradation	Exceptionally strong |S11| peak, significant |S21| attenuation	Brittle fracture entirely along welding interface or void zone	Mandatory resection and reconstruction

## Data Availability

No new data were created or analysed in this study. Data sharing is not applicable to this article.
